# Leveraging graph-based hierarchical medical entity embedding for healthcare applications

**DOI:** 10.1038/s41598-021-85255-w

**Published:** 2021-03-12

**Authors:** Tong Wu, Yunlong Wang, Yue Wang, Emily Zhao, Yilian Yuan

**Affiliations:** Advanced Analytics, IQVIA Inc., Plymouth Meeting, PA USA

**Keywords:** Health care, Computational science

## Abstract

Automatic representation learning of key entities in electronic health record (EHR) data is a critical step for healthcare data mining that turns heterogeneous medical records into structured and actionable information. Here we propose ME2Vec, an algorithmic framework for learning continuous low-dimensional embedding vectors of the most common entities in EHR: medical services, doctors, and patients. ME2Vec features a hierarchical structure that encapsulates different node embedding schemes to cater for the unique characteristic of each medical entity. To embed medical services, we employ a biased-random-walk-based node embedding that leverages the irregular time intervals of medical services in EHR to embody their relative importance. To embed doctors and patients, we adhere to the principle *“it’s what you do that defines you”* and derive their embeddings based on their interactions with other types of entities through graph neural network and proximity-preserving network embedding, respectively. Using real-world clinical data, we demonstrate the efficacy of ME2Vec over competitive baselines on diagnosis prediction, readmission prediction, as well as recommending doctors to patients based on their medical conditions. In addition, medical service embeddings pretrained using ME2Vec can substantially improve the performance of sequential models in predicting patients clinical outcomes. Overall, ME2Vec can serve as a general-purpose representation learning algorithm for EHR data and benefit various downstream tasks in terms of both performance and interpretability.

## Introduction

Recent years have seen an explosive growth of electronic health record (EHR) data, which has motivated extensive use of machine learning methods, in particular deep learning, in tasks such as diagnosis prediction^1,2^, risk prediction^3,4^, and patient subtyping^5,6^. Under the hood, all these tasks involve some form of neural networks that learn features or patterns from data, a task often referred to as *representation learning*.

**Figure 1:**
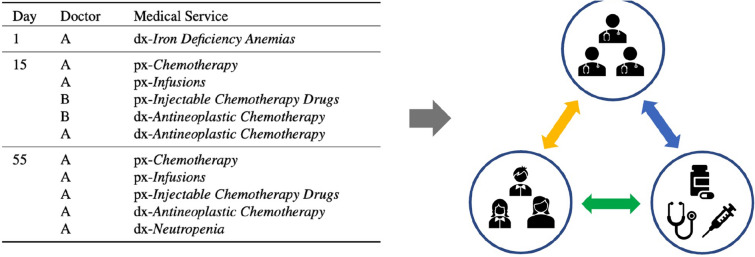
Illustration of patient medical journey where ‘dx’ and ‘px’ represent diagnosis and medical procedure, respectively, and its conversion into a graph. The arrows with different colors denote different types of relations between medical entities.

One major challenge of representation learning in EHR comes from the heterogeneity of the various medical entities that compose EHR data, including diagnoses, prescriptions, lab test results, medical procedures, doctor profiles, and patient demographics, etc., that are a mixture of tabular values, text notes, and medical codes. In addition, medical entities of different types can form complex relations between each other. As illustrated in the table of Fig. 1, a patient may visit one or more clinical sites multiple times with irregular time intervals, with each visit generating a varying number of medical service records (diagnoses, prescriptions, or procedures) from possibly different doctors. Various machine learning or deep learning techniques, including recurrent neural network (RNN)^7,8^, convolutional neural network (CNN)^9,10^, Restricted Boltzmann Machine (RBM)^11,12^, autoencoder^13,14^, and many more, have been employed for many EHR based learning tasks. Despite the made progress, they are usually limited to emphasize on single and homogeneous aspects of EHR data, such as sequence of medical codes or bag-of-words representation of medical histories, to do the task-specific modeling, while leaving the rich amount of interactions between different types of medical entities underexploited. For example, patients sharing the same doctors on multiple medical treatments tend to have similar profiles of disease progression, which can be leveraged for improved diagnosis prediction and risk stratification^15,16^. However, integrating the patient-service and patient-doctor relations simultaneously is nontrivial and difficult under the frameworks of conventional structures such as CNN or RNN.

The versatile information embedded in the EHR tables is characterized by a rich level of relationship (e.g., patients to doctors, doctors to services, and patients to services) that can be naturally and accurately captured in a graph-based data structure. As illustrated in the diagram of Fig. 1, the medical graph created from EHR consists of three clusters of nodes representing different entities (patients, doctors, and medical services) as well as edges connecting nodes that denote intra-cluster or inter-cluster relations. Therefore, how to model EHR data with graphs and learn informative and effective representations of medical entities from graphs are of great interest and in increased demand by researchers and practitioners from academics and healthcare industries^17–21^. Candidate graph representation learning approaches include a number of graph embedding algorithms that are developed for processing generic graphs, such as DeepWalk^22^, node2vec^23^, GraRep^24^, HOPE^25^, and LINE^26^. They are unsupervised methods with the objective to maximally preserve network structures by either predicting neighbors in the paths generated from random walks over graphs (e.g., DeepWalk, node2vec), or maintaining the first- and second-order (or even higher orders) neighborhood topologies of nodes in graphs (e.g., LINE). There are also supervised graph embedding algorithms that typically leverage graph neural networks (GNN) to preserve not only graph structures but also node/edge properties when such extra side information is available, such as GraphSAGE^27^ and Graph Attention Network (GAT)^28^. However, these established methods face limitations in applications involving EHR data due to (1) they are developed for generic graphs, hence are sometimes insufficient to address niche characteristics of medical graph applications; (2) they commonly lack the mechanism to properly handle temporal sequences that is a hallmark of EHR data where all medical services are timestamped. To better utilize niche characteristics of EHR data, Choi et al. proposed Med2Vec^29^ and Graph Convolutional Transformer (GCT)^30^ that leveraged the explicit or implicit categorization of diagnosis codes over treatment codes in each encounter to learn multilevel medical embedding in healthcare prediction tasks. Though effective, their approaches do not consider the temporal characteristics unique to individual medical services, hence cannot address the irregular time intervals of encounters in patient journeys.

To address the ubiquitous situation that real-world networks are composed of nodes of different types, heterogeneous network embedding algorithms have been proposed, including metapath2vec^31^, HNE^32^, CAHNE^33^, MARU^34^, and more. These methods can be borrowed to represent the versatile information contained in medical graphs. A setting shared by these methods is the definition of heterogeneous networks as a collection of nodes of several unique types and edges connecting the nodes. However, a common limitation of these heterogeneous network embedding methods is the lack of flexibility of representing edges of different types, which can take completely different forms, as nodes of different types may interact in fundamentally different ways. For example, as will be detailed later, we believe that the relations of patient-service and doctor-service are of different types and should be treated with separate modeling mechanisms.

In this work, we propose a graph-based, hierarchical medical entity embedding framework ME2Vec to address the aforementioned challenges. ME2Vec features a hierarchical structure that embeds medical services first, then doctors, patients at last, such that we can employ the most suitable embedding mechanism catered for each specific type of entity while utilizing niche characteristics of EHR data. At the service level, we propose to characterize the importance of various medical services with their co-occurrence frequencies. Concretely, important services are typically infrequent in patient journeys, hence their co-occurrence frequencies with other services are smaller than those of routine services. With a proximity-preserving embedding approach (e.g. node2vec^23^), important services with small co-occurrence frequencies will be far away from other services in the embedding space, thus emphasizing their importance via “spatial isolation”. At the doctor and patient levels, a fundamental principle that we adhere to is “*it’s what you do that defines you*”, which empowers the interpretability of embeddings. Following this principle, a doctor’s embedding should characterize the doctor’s primary specialty (e.g., neurologist or oncologist). We propose an auxiliary task that predicts a doctor’s specialty (available in EHR) from the embeddings of medical services performed by the doctor, thereby the doctor embedding can be optimized. To preserve the network proximity of patients with respect to doctors and services, we develop a method called *duplication & annotation* that converts an attributed multigraph to a simple graph without loss of structural information, over which efficient and scalable graph embedding techniques can be applied to obtain patient embedding.

Overall, ME2Vec provides a comprehensive solution of embedding medical entities, thus can serve as a general-purpose representation learning algorithm for EHR data. The source code of ME2Vec is provided at https://github.com/tong-wu-umn/ME2Vec.

## Methods

### Data sources

Data were extracted from proprietary IQVIA US longitudinal database. This real-world database contains US longitudinal prescription (Rx) and medical claims (Dx). The database captures the complete patient journey for all services billed to and covered by the patient’s health plan, where patients are from commercial plans with medical and pharmacy benefits. Rx data are collected from retail and mail pharmacies, as well as long term care facilities. They track what therapy a patient starts on and how it changes over time. This information is critical in tracking behaviors over time, measuring product success, understanding challenges, and making well-informed decisions around new opportunities. Dx data are collected from services performed in the physician’s office. They include patient level diagnosis, procedures and in-office treatments for visits to U.S. office-based professionals, ambulatory and general healthcare sites. Dx data are critical in understanding patient diagnoses and in-office procedures and drug administrations. They track what takes place during a patient’s visit with their physician. Knowing what conditions a patient is diagnosed with, what procedures are performed, and what drugs are administered is important in understanding why therapies are started or changed.

IQVIA collects and links the health data anonymized by a proprietary, automated deidentification engine that encrypts and removes personal health information (PHI) properly. Informed consent is waived as data anonymization happened prior to being collected by IQVIA. The deidentification process is the Health Insurance Portability and Accountability Act (HIPAA) compliant certified and Institutional Review Board exempt. Throughout all the data processing and analyses in our studies, the health data maintained deidentified by strictly following necessary measures to avoid re-identification. The approving body of all the experimental protocols in this work is the IQVIA’s internal Privacy and Legal Auditing Team. All the methodologies described in this work were carried out in accordance with the relevant guidelines and regulations.

To enhance the reproducibility of the proposed method and facilitate fair and easy comparison with others, we have also included the evaluation of the performance of ME2Vec and several baselines on a public dataset, the eICU Collaborative Research Database^35^, that comprises deidentified health data associated with over 200,000 patient unit encounters for over 139,000 unique patients admitted to one of 335 units at 208 hospitals in the United States between 2014–2015. The eICU dataset includes vital sign measurements, care plan documentation, severity of illness measures, diagnosis and treatment information. All tables are deidentified to meet the safe harbor provision of the US HIPAA. These provisions include the removal of all protected health information. Hospital and unit identifiers have also been removed to protect the privacy of contributing organizations. The schema was established in collaboration with Privacert (Cambridge, MA), who certified the re-identification risk as meeting safe harbor standards (HIPAA Certification no. 1031219-2).

### Service embedding

From a similarity perspective, patients receiving the same medical services tend to be similar in terms of medical conditions. However, two patients are not necessarily similar simply because they all have *hypertension*, as their hypertension could be comorbidities of different and more severe medical conditions. In other words, routine services (e.g., *hypertension* or *blood counts measurement*) should be considered with less importance when evaluating patient similarity compared with more complicated services which are typically infrequent in patient journeys.

In word2vec, the distance between two word embeddings reflects their co-occurrence frequency derived from a text corpus. Similarly, we can estimate the co-occurrence frequency of every pair of medical services by using a fixed-size context window from patient journeys. In analogy to words in a document following a semantic and grammatical order, the sequence of medical services in a patient journey is jointly determined by the patient’s disease progression and doctors’ treatment decisions. By applying contextual embedding algorithms, we can derive service embeddings that preserve the inter-service distances, wherein a small co-occurrence frequency corresponds to long distance, and vice versa. Therefore, we can denote the “importance” of services towards evaluating patient similarity by inspecting their geographical distributions in the embedding space, where a “lonely” service node is weighted more importantly than nodes crowding together.

We first create the graph of medical services ${G}_{svc}=({S}, {E}_{svc})$, where ${S}=\{s_1, s_2, \dots , s_{|{S}|}\}$ is the set of medical services, and ${E}_{svc}$ is the set of edges connecting medical services. The weight of $e_{ij}$ denotes the co-occurrence frequency of services $s_i$ and $s_j$, and also the element of the adjacency matrix ${\mathbf {A}}_{svc} \in {\mathbb {R}}^{|{S}| \times |{S}|}$ at (*i*, *j*). To obtain ${\mathbf {A}}_{svc}$, we use a context window spanning *T* days to traverse all patient journeys with no overlap. Using a fixed number of days allows a flexible number of services per context window and guards against “service explosion” that many medical services are administered in one day but are separated into different context windows. We update ${\mathbf {A}}_{svc}$ with the count of the occurrence of each unique pair of medical services appeared within the *T* days of the context window by it to the corresponding element of ${\mathbf {A}}_{svc}$. Note that (1) the co-occurrence frequencies from different patients are summed together, thus reflecting a generalized knowledge of the time intervals between medical services, which can enhance the transferability and privacy of the learned service embedding; (2) the choice of *T* also serves as a proxy to control the sparseness of ${\mathbf {A}}_{svc}$: a smaller *T* will lead to a sparser ${\mathbf {A}}_{svc}$, and vice versa.

As we are interested in preserving temporal distances between medical services, a biased-random-walk-based embedding scheme is a better choice than word2vec or DeepWalk^22^, because it can allow for more accurate estimation of a node’s location in a graph through biased random walks by generating “pseudo sequences”, wherein service nodes of higher degree appear more frequently. In this work, we adopted node2vec^23^ in service embedding as it can provide extra tunable parameters to adjust redundant node sampling and also balance breadth-first and depth-first search. The details of service embedding are given in Supplementary Algorithm 1.

### Doctor embedding

We observe that medical services administered by a doctor exhibit patterns that are consistent with the doctor’s primary specialty. For example, medications and/or medical procedures administered by an *obstetrician* (or *gynecologist*) are in general different from those of an *oncologist*. This inspires us to train doctor embedding in an auxiliary task by predicting a doctor’s primary specialty from the doctor’s medical services recorded in EHR. The auxiliary task is supervised as we can leverage the primary specialty information that is normally available in patient journeys. Another practical benefit of the supervised learning formulation is that we can reuse the learned classification model to predict missing doctor specialties (which is common in many medical databases) based on their administered medical services.

To account for that doctor embedding should reflect the specific medical services of each doctor, we initialize the embedding of a doctor as the weighted average of the embedding vectors of the medical services conducted by the doctor, such that the trained doctor embedding can be close to its associated medical services in the embedding space. As the amount and type of unique medical services vary significantly for different doctors, we propose to use Graph Attention Network (GAT)^28^ to predict doctor specialties from services, as the attention mechanism naturally supports mapping from a varying number of inputs to output.

For a doctor $d_j$ whose conducted medical services are $\{s_i\}^{(d_j)}$, the attention coefficient $e_{ij}$ between the doctor embedding ${\mathbf {d}}_j$ and each of the service embeddings $\{{\mathbf {s}}_i\}^{(d_j)}$ conducted by doctor $d_j$ is

1 $$\begin{aligned} e_{ij} = {\texttt {LeakyReLU}}\left( {\mathbf {a}}^T[{\mathbf {Wd}}_j||{\mathbf {Ws}}_i]\right) , \end{aligned}$$

where $\{{\mathbf {d, s}}\} \in {\mathbb {R}}^p$, ${\mathbf {a}} \in {\mathbb {R}}^{2p^{\prime }}$, ${\mathbf {W}} \in {\mathbb {R}}^{p^{\prime } \times p}$, LeakyReLU is the Leaky Rectified Linear Unit with a negative input slope of 0.2^36^, $\cdot ^T$ represents transposition, and $\parallel$ is the concatenation operation. $\{{\mathbf {W, a}}\}$ are parameters of the aggregation functions that “aggregate” the information of neighboring service vertices into the targeted doctor vertex. After normalizing the attention coefficient through a softmax layer, we obtain the final expression:

2 $$\begin{aligned} \alpha _{ij} = \frac{\text {exp}\left( {\texttt {LeakyReLU}}({\mathbf {a}}^T[{\mathbf {Wd}}_j||{\mathbf {Ws}}_i])\right) }{\sum _{s_k \in {N}_{d_j}}\text {exp}\left( {\texttt {LeakyReLU}}({\mathbf {a}}^T[{\mathbf {Wd}}_j||{\mathbf {Ws}}_k])\right) }. \end{aligned}$$

where $N_{d_j}$ represents the collection of services administered by doctor $d_j$.

The updated embedding vector of doctor $d_j$ can then be obtained as a linear combination of the associated service embeddings weighted by corresponding attention coefficients. To stabilize the learning process, we adopt a multi-head attention comprising *K* heads. The operation of the multi-head attention layer can be described as

3 $$\begin{aligned} {\mathbf {d}}_j^{\prime } = \mathop {\parallel }\limits _{k=1}^K \sigma \left( \sum _{s_i \in {\mathcal {N}}_{d_j}}\alpha _{ij}^k{\mathbf {W}}^k{\mathbf {s}}_i\right) . \end{aligned}$$

Note that we have already obtained ${\mathbf {s}}_i$ in service embedding, thus making the doctor embedding a simper task than ordinary graph embedding wherein the embeddings of all nodes are unknown and to be learned.

We first create a bipartite graph ${G}_{doc}$ consisting of two sets of vertices, doctors ${D}=\{d_1,d_2,\dots , d_{|{D}|}\}$ and medical services ${S}=\{s_1, s_2, \dots , s_{|{S}|}\}$. ${E}_{doc}$ is the set of edges connecting the two sets of vertices, where the weight of each edge represents the number of times that doctor $d_j$ has administered service $s_i$. Next, we initialize the embedding vector of each doctor vertex as the weighted average of the embedding vectors of its connected service vertices. After that, we update the doctor embeddings using equations (2) and (3) by predicting the primary specialty of each doctor. Finally, we obtain the aggregation functions parameterized by $\{{\mathbf {W}}^k, {\mathbf {a}}^k\}_{k=1}^K$ that can be used to derive embeddings of not only doctor vertices already in the patient journeys, but also new doctor vertices that might be added in the future. The details of doctor embedding are given in Supplementary Algorithm 2.

### Patient embedding

**Figure 2:**
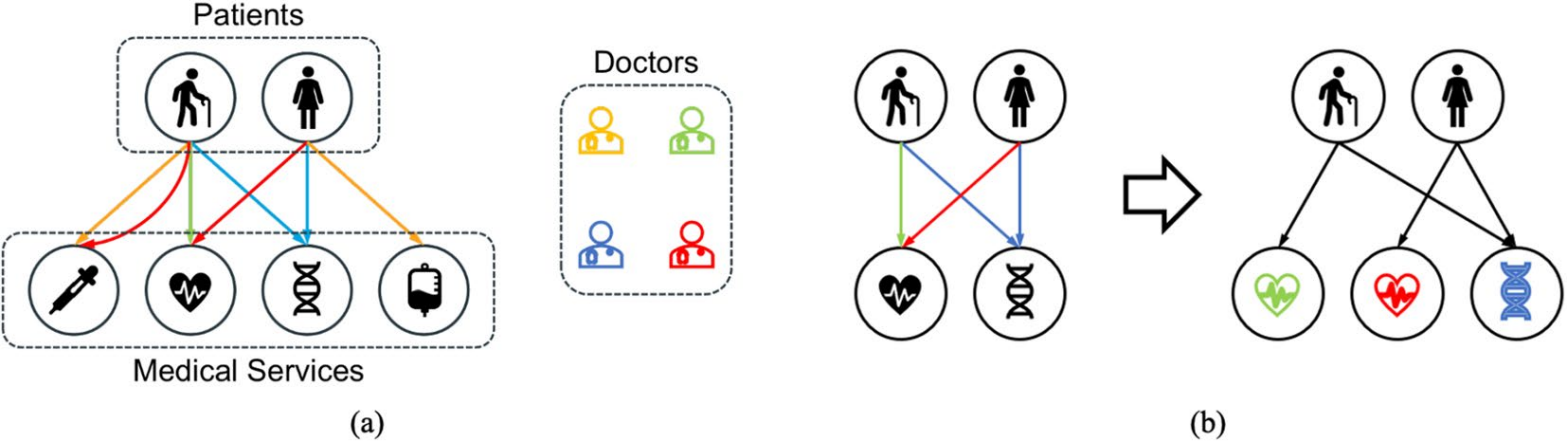
(**a**) A toy example illustrating the complex similarity relations of patients with both doctors and medical services. An edge denotes the patient has received the service to which the edge connects. The color of an edge denotes the particular doctor who administered that service. (**b**) A toy example illustrating the *duplication and annotation* in patient embedding. After *duplication*, one new ECG service node is generated; after *annotation*, all service nodes are annotated with their edge attributes, and edges have no doctor attributes but weights.

The similarity between patients can be defined from the perspectives of shared doctors and/or shared services. In general, we expect the patient embedding can facilitate that *patients are more similar to each other if they receive the same medical services from the same doctors.* Following this guideline, patient similarity can be categorized into: (1) different patients receive the same services from the same doctor; (2) different patients receive the same services from different doctors; (3) different patients receive different services from the same doctor; (4) one patient receives the same service multiple times from different doctors. We illustrate these scenarios in Fig. 2a, where an arrow indicates a patient (starting node) has received a service (ending node) from a doctor (the color of the edge).

The versatile forms of patient similarity can be formalized as a *bipartite multigraph* ${G}_{pat}$, where the two disjoint sets of vertices (*P* and *S*) represent the patients and services, respectively. A multigraph allows multiple edges connecting two nodes, which precisely models the scenario that a patient may have received the same service multiple times from different doctors. An edge connecting patient $p_k$ and service $s_i$ carries two attributes: the doctor $d_j$ who treated $p_k$ with $s_i$, and the weight $w_{p_k \rightarrow d_j \rightarrow s_i}$ denoting the count of the service. The challenge of embedding patient vertices in a bipartite multigraph comes mainly from the attributed edges. In an attributed network, the node or edge attributes are often heterogeneous with respect to the network structure, thus creating difficulty in joint information extraction. A common practice of recent efforts to address this challenge is to generate heterogeneous “meta-paths” that consist of both entity nodes and their attribute nodes by random walks, followed by complex deep learning models to learn the node embeddings^37,38^. Though being effective, it raises a concern about the efficiency of a stochastic random walker exploring the network structure, especially in a multigraph where multiple edges with different attributes connect a pair of nodes that demands more extensive localized searches of a node’s neighborhood. Furthermore, random-walk-based embeddings generalize node connections beyond existing network topologies, which would potentially result in more false alarm predictions and hence must be used with extra cautions on the patient level.

In this work, we propose a simple and scalable node embedding algorithm tailored for attributed multigraph. Our algorithm is an extension of the network embedding algorithm LINE^26^. First, we develop an approach called *duplication & annotation* to convert ${G}_{pat}$ into a simple graph with no attributes:

- *Duplication:* We duplicate each service node by the number of unique attributes of the edges linked to the node. A service node will not be duplicated if all its edges are of the same attribute. For example, as shown in Fig. 2b, the electrocardiography (ECG) service has two edges with two different doctor attributes, thus was duplicated into two new ECG nodes, whereas the gene service has two edges with the same doctor attribute, and was not duplicated. After duplication, a service node must connect to either multiple edges with the same attribute or a single attributed edge.
- *Annotation:* We annotate each service node with the doctor attribute of its edges into a “hybrid node”, and remove the doctor attribute from its edges, thereby converting a multigraph into a simple graph with no attributed edges.

*Annotation* can be implemented as a linear transformation of the concatenated doctor and service embedding vectors (note at this point we have already obtained the doctor and service embedding vectors):

4 $$\begin{aligned} {\mathbf {h}}_{s_i,d_j} = {\mathbf {W}}_{a}[{\mathbf {s}}_i || {\mathbf {d}}_j]+{\mathbf {b}}_{a}, \end{aligned}$$

where ${\mathbf {W}}_{a} \in {\mathbb {R}}^{p^{\prime \prime } \times (p+p^\prime )}$, ${\mathbf {b}}_{a} \in {\mathbb {R}}^{p^{\prime \prime }}$, and ${\mathbf {h}}_{s_i,d_j} \in {\mathbb {R}}^{p^{\prime \prime }}$ is the embedding of the hybrid node created from $s_i$ and $d_j$.

In LINE, node embeddings are optimized by preserving nodes’ first-order and second-order proximities defined in the network structure. As in patient embedding, we are dealing with a bipartite graph, and that the embedding vectors of the hybrid nodes are already known (except for the transformation parameters), we can skip the first-order part and focus on optimizing the second-order proximities of patient nodes only. For a patient $p_k$, its second-order proximity relative to other patients is defined over the “context” probability of seeing a hybrid node $h_{s_i, d_j}$:

5 $$\begin{aligned} p_2(h_{s_i, d_j}|p_k)=\frac{\text {exp}({\mathbf {h}}_{s_i,d_j} \cdot {\mathbf {p}}_k)}{\sum _{l \in \{h\}}\text {exp}({\mathbf {h}}_l \cdot {\mathbf {p}}_k)}, \end{aligned}$$

where ${\mathbf {p}}_k \in {\mathbb {R}}^{p^{\prime \prime }}$ and $\{h\}$ is the collection of all hybrid nodes. Meanwhile, each context probability $p_2$ corresponds to an empirical distribution defined by the edge weights:

6 $$\begin{aligned} {\hat{p}}_2(h_{s_i, d_j}|p_k)=\frac{w_{p_k \rightarrow h_{s_i,d_j}}}{\sum _{l \in {N}_{p_k}}w_{p_k \rightarrow h_l}}, \end{aligned}$$

where ${N}_{p_k}$ represents the collection of all hybrid node neighbors of patient $p_k$. Then we can optimize $\{{\mathbf {p}}_k\}_{k=1}^P$, ${\mathbf {W}}_{a}$, and ${\mathbf {b}}_{a}$ by minimizing the following loss function

7 $$\begin{aligned} {L}_{pat}=\sum _{k=1}^P D_{KL}\left( {\hat{p}}_2(\cdot |p_k), p_2(\cdot |p_k) \right) , \end{aligned}$$

where $D_{KL}$ is the *Kullback–Leibler* (*KL*) distance. Plugging Eq. (6) into (7) and expanding the *KL* distance, we have

8 $$\begin{aligned} {L}_{pat} = -\sum _{(i, j, k) \in {E}_{pat}} \frac{w_{p_k \rightarrow h_{s_i, d_j}}}{\sum _{l \in {N}_{p_k}}w_{p_k \rightarrow h_l}}\text {log}(p_2(h_{s_i,d_j}|p_k)), \end{aligned}$$

where ${E}_{pat}$ is the set of all edges of the patient-service bipartite graph after *duplication & annotation*. The details of patient embeddings are given in Supplementary Algorithm 3.

## Experiments and results

### Cohort preparation

We selected patients diagnosed with chronic lymphocytic leukemia (CLL) between January 2012 and December 2018 from the IQVIA database and discarded patients whose Rx/Dx records are incomplete. The remaining patients were used as the positive cohort. For each patient in the positive cohort, we pulled one-year clinical records six months before the date of the first CLL diagnosis. To prepare the negative cohort, we extracted all the patients between January 2018 and December 2018 from the IQVIA database who have shown CLL risk factors and related symptoms but without a CLL diagnosis. In both the positive an negative cohorts, we kept patients who are greater than 18 years old. The criteria for choosing patients into the negative cohort are provided in Supplementary Section B.1. Some important statistics of the extracted dataset are listed in Table 1.

**Table 1:** Statistics of the proprietary IQVIA dataset.

Number of total patients	8942
Number of CLL patients	1241
Number of non-CLL patients	7701
Number of doctors	8170
Number of unique doctor primary specialties	114
Number of unique medical services	394
Average number of services per patient	111
Average number of unique services per patient	11.09
Average number of unique doctors per patient	1.54
Average number of unique doctor specialties per patient	1.33

For the eICU dataset, we predicted readmission to ICU based on patients’ medical records including diagnoses, prescriptions, and medical procedures in the current encounter, while excluding lab test results and vital sign measurements that are not available in claim data, such that the data configuration is consistent with that of the IQVIA dataset. Unlike the IQVIA dataset where the doctor information (ID and specialty) is available for each recorded medical service, the eICU dataset has only one managing physician recorded for an entire admission that could consist of up to hundreds to thousands of medical services. As normally patients are admitted into ICUs due to certain urgent medical conditions (e.g., heart failure) and receive treatments specific to their conditions, we considered the specialty of managing physicians as that of all the medical services recorded in one admission as an approximation. In addition, we limited the patient cohort to those who have at least 5 medical services recorded in one admission. Eventually, we extracted 141,666 encounters for 110,910 patients from the eICU dataset. Table 2 lists some important statistics of the extracted data.

**Table 2:** Statistics of the eICU dataset.

Number of total patients	110,910
Number of encounters (admissions)	141,666
Number of ending encounters (positive readmission)	18,983
Number of non-ending encounters (negative readmission )	122,783
Number of unique specialties of managing physicians	49
Number of unique medical services	3157
Average number of admissions per patient	1.24
Maximum number of admissions per patient	24
Average number of services per encounter	43.8
Minimum number of services per encounter	5
Maximum number of services per encounter	11,696

### Experimental setup

We compared ME2Vec with the following baselines for medical entity embedding: node2vec^23^, LINE^26^, spectral clustering (SC)^39^, non-negative matrix factorization (NMF)^40^, and metapath2vec^31^. The baselines for comparison cover a wide range of types of representation learning approaches: SC and NMF are traditional methods based on matrix factorization; node2vec and metapath2vec are random-walk based graph embedding methods; LINE is also a graph embedding method by maximally preserving the first and second-order neighbors of each node. Among them, metapath2vec is a recent competitive heterogeneous network embedding algorithm that can learn latent vectorized representations for nodes/edges of various types simultaneously. It is based on node2vec with the extra constraint on random walks that must hop between nodes/edges of different types in certain user-specified orders (so called “meta paths”). In this work, we set the meta path as $patient \rightarrow doctor \rightarrow service \rightarrow doctor \rightarrow patient$ for the IQVIA dataset; for the eICU dataset, the *doctor* nodes in the meta path were replaced as *specialty*. With the specified meta paths, the topology of the heterogeneous network comprising different medical entities can be thoroughly explored.

In all the experiments below, we used the Adam optimizer^41^ to update the parameters of ME2Vec and LINE. Also, the amount of negative samples in training the graph embedding based methods (ME2Vec, LINE, node2vec, and metapath2vec) is set as 10. For ME2Vec, the context window length *T* is set as 8 days for the IQVIA dataset and 60 (1 h) for the eICU dataset, and the number of attention heads *K* is 4. The dimensions of embeddings for all entities are set as 128. Detailed parameter settings can be found in the source code available at the authors’ GitHub repo.

### Embedding visualization

**Figure 3:**
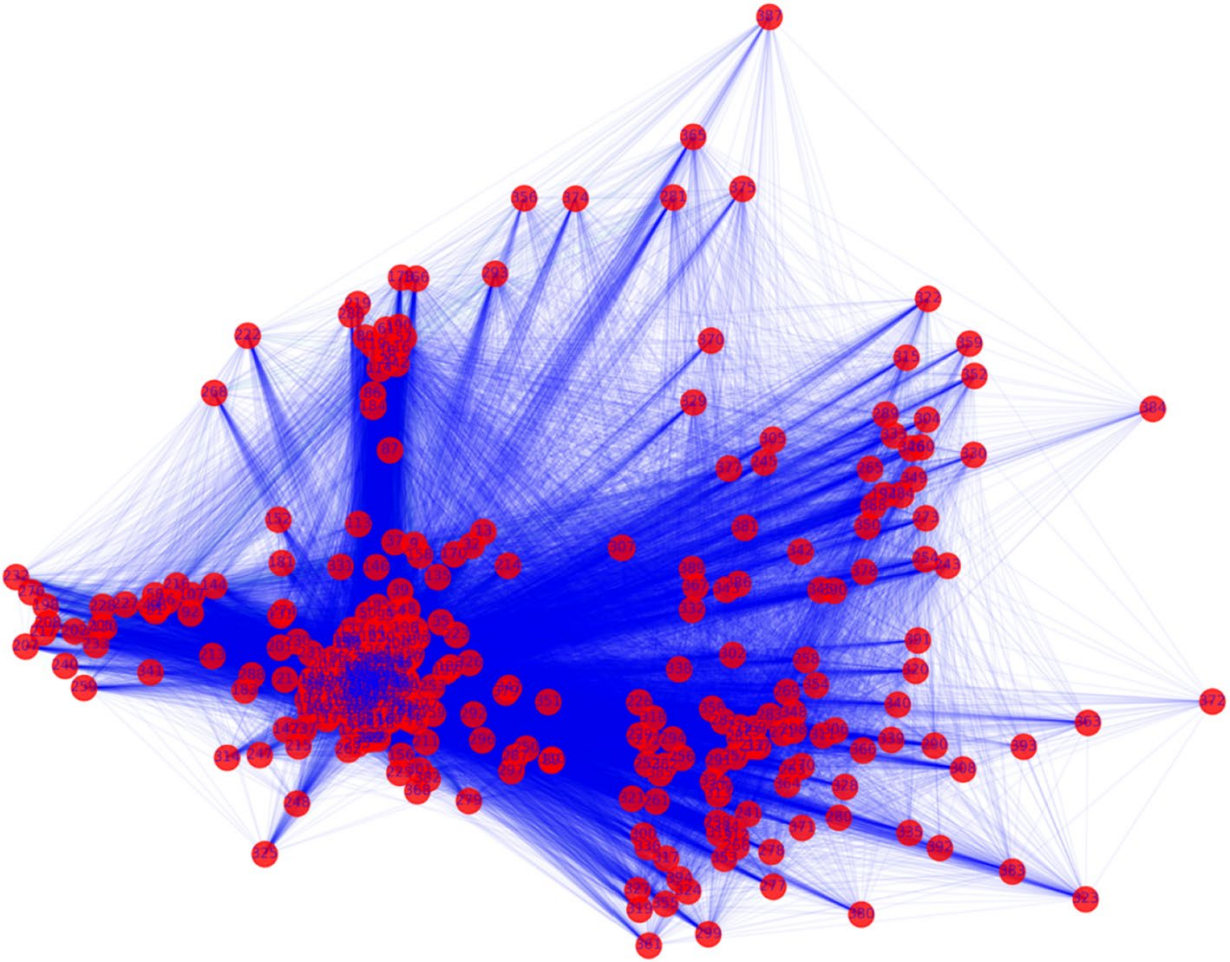
2-dimensional visualization of service embeddings from ME2Vec after PCA. Each red dot represents a medical service with its ID labeled. Each blue line connecting two dots indicates that the two services co-occur as least once.

We visualize the trained embedding vectors of 394 medical services in Fig. 3. The 128-dimensional vectors are projected to a 2-dimensional space via principal component analysis (PCA). Figure 3 shows clearly that infrequent services (with larger IDs) spread out in the embedding space, whereas routine services (with smaller IDs) aggregate themselves closely in the centering area, which ensures the “spatial isolation” of important medical services.

**Figure 4:**
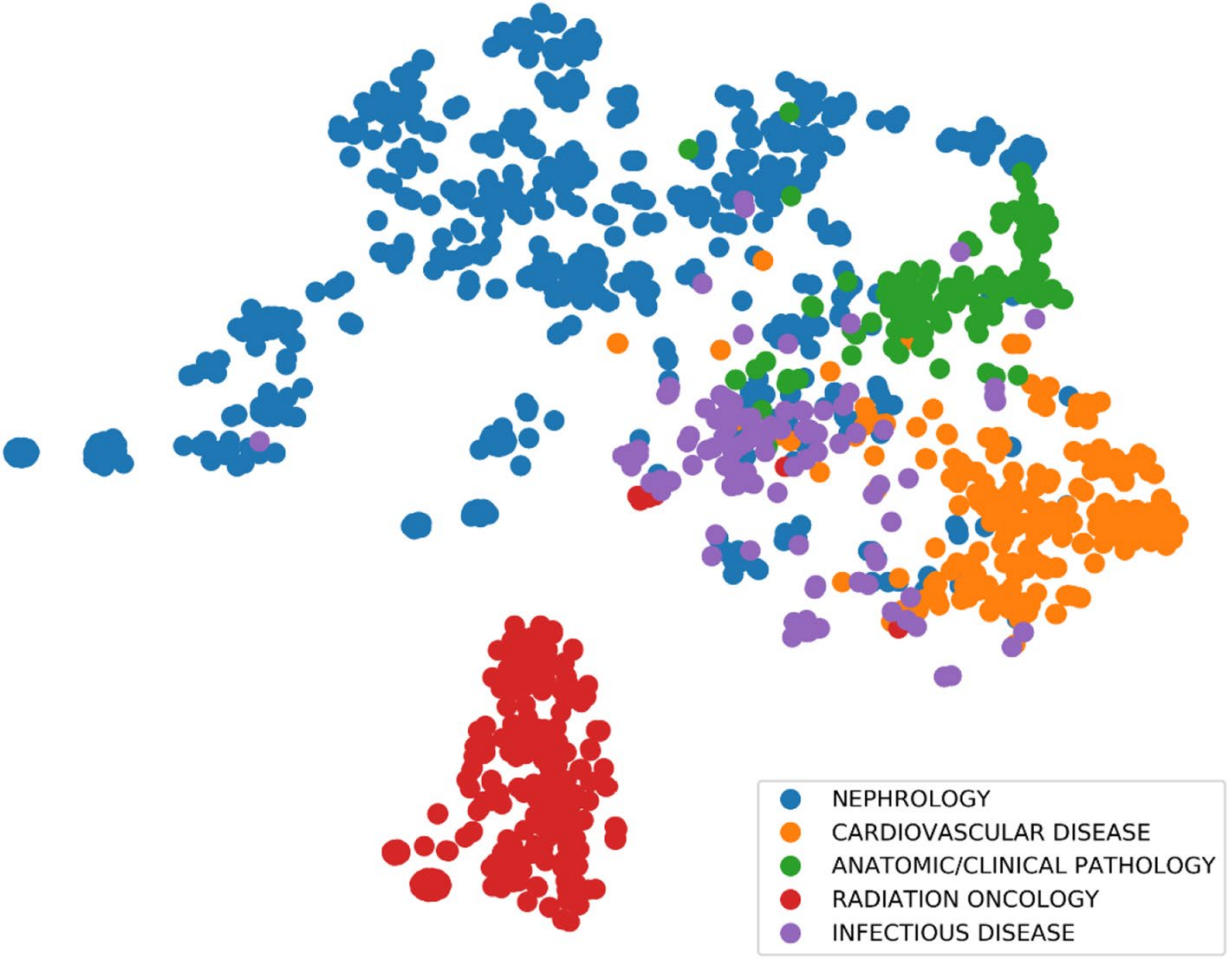
2-dimensional visualization of a portion of doctor embeddings from ME2Vec after t-SNE. Each dot represents a doctor, with its color indicating the doctor’s primary specialty. Doctors with five different primary specialties are displayed for illustration.

We also visualize the trained embedding vectors of some of the doctors in Fig. 4, where we can see a clear separation of doctors with different primary specialties. For example, *nephrology* doctors are far away from *cardiovascular disease* doctors, while *radiation oncology* doctors are even further away from the rest. This result also verifies our hypothesis that medical services administered by doctors exhibit patterns that can be leveraged to categorize doctors into clusters corresponding to their specific specialties. In practice, the graph attention model used to learn doctor embedding can also be adopted to infer missing doctor primary specialties, which is common in many EHR databases.

### Node classification

We first trained ME2Vec and the baselines on the entire dataset to obtain patient embeddings for each of the methods. Except metapath2vec, the baseline methods cannot integrate information from both doctors and services at the same time. To address this, we created two bipartite graphs from the dataset that model the patient-doctor and patient-service relations, respectively. Therefore each baseline has two versions of patient embeddings, with one derived from the patient-service graph, and the other derived from the patient-doctor graph.

Next, we used the patient embeddings in the training set as well as their diagnostic labels to train a logistic regression (LR) classifier with L2 regularization. After that, we predicted the diagnostic labels of patients in the testing set from their embeddings using the trained LR classifier. We varied the training ratio from 20% to 80%, and under each training ratio we repeated the experiment for 10 times with randomized train/test split and reported the average micro-F1 and macro-F1 in Table 3. The results show that ME2Vec outperforms all baselines. It is worth noting that all baselines achieve consistently poorer performance from the patient-doctor graph than from the patient-service graph, suggesting their common weakness of extracting useful information from the patient-doctor relation. Additionally, for each baseline, we tried a simple integration by concatenating the two versions of patient embeddings, which, however, did not lead to consistent performance improvement, and hence not reported.

**Table 3:** Performance of node classification in micro-F1 and macro-F1.

Algorithms	Micro-F1				Macro-F1			
	20%	40%	60%	80%	20%	40%	60%	80%
ME2Vec	**0.869**	**0.877**	**0.878**	**0.879**	**0.664**	**0.679**	**0.682**	**0.676**
metapath2vec	0.865	0.868	0.869	0.870	0.522	0.551	0.574	0.577
node2vec (service)	0.865	0.875	0.876	0.878	0.613	0.630	0.632	0.640
node2vec (doctor)	0.850	0.862	0.860	0.861	0.474	0.466	0.462	0.463
LINE (service)	0.855	0.864	0.866	0.866	0.587	0.592	0.592	0.586
LINE (doctor)	0.854	0.863	0.860	0.861	0.470	0.465	0.462	0.463
SC (service)	0.862	0.861	0.861	0.868	0.463	0.463	0.463	0.465
SC (doctor)	0.862	0.861	0.861	0.868	0.463	0.463	0.463	0.465
NMF (service)	0.868	0.870	0.869	**0.879**	0.584	0.586	0.589	0.600
NMF (doctor)	0.861	0.860	0.860	0.867	0.469	0.472	0.470	0.469

Bolded numbers indicate best performance compared with the rest in the column.

### Link prediction

In this experiment, we predict if a patient should see a doctor. This task has a direct real-world significance that we can leverage the trained medical entity embeddings for personalized physician targeting. We first randomly removed 10% of the edges from the patient-doctor graph as the positive edges, while ensuring that the residual graphs are still connected. For the negative edges, we randomly sampled an equal amount of node pairs from the original patient-doctor graph, which have no edges connecting them. We then obtained the embeddings of patients and doctors using ME2Vec and the baselines from the residual graphs, and trained an LR classifier for each method to predict edge existence between a patient-doctor node pair. The input to the LR is the concatenation of two embeddings. We reported the performance in area-under-the-curve of (AUC) as shown in Table 4.

**Table 4:** Performance of link prediction in AUC.

	metapath2vec	ME2Vec	node2vec	LINE	NMF	SC
AUC	0.758	0.736	0.608	0.552	0.521	0.508
Improvement	0%	+3.0%	+24.7%	+37.3%	+45.5%	+49.2%

**Table 5:** Performance of readmission prediction in AUC and PR-AUC.

	ME2Vec	metapath2vec	node2vec	LINE	SC	NMF
PR-AUC	0.186	0.183	0.181	0.178	0.177	0.164
Improvement	0%	+1.64%	+2.76%	+4.49%	+5.08%	+13.41%
AUC	0.588	0.576	0.573	0.570	0.560	0.524
Improvement	0%	+2.08%	+2.62%	+3.16%	+5.00%	+12.21%

On predicting patient-doctor edges, metapath2vec achieved the highest AUC, followed by ME2Vec and node2vec. In general, the performance of graph-based embedding methods was significantly better than matrix factorization based methods. The superior performance of metapath2vec was most likely thanks to the meta path of random walks that explicitly explored and reinforced the relationship from medical services to doctors, and that from doctors to patients. Meanwhile, the advantage of ME2Vec on predicting patient-doctor edges can be attributed to the hierarchical structure where doctor embeddings are defined over associated service embeddings, hence encoding a richer amount of information and leading to superior predictability. In comparison, the doctor embeddings from the rest baselines are solely derived from their structural proximities to the patients.

### Readmission prediction

In this task, we used patients’ medical records in the eICU dataset including diagnoses, prescriptions, and procedures as well as the primary specialties of managing physicians to predict if the patients will be readmitted into ICUs in the future. Patients who are readmitted to ICUs tend to have increased length of stay, healthcare expenditure, and mortality compared to those who are never readmitted. Improving risk stratification for patients after ICU discharge could have important benefits for critically ill hospitalized patients^42^.

The preprocessing of the eICU dataset for prediction modeling was similar to that of the node classification task, except that for SC and NMF, we only considered the patient-service graph as there are only 49 unique specialties over which matrix factorization based dimensionality reduction is not viable as the original dimension (49) is already smaller than the expected dimension (128). For ME2Vec, metapath2vec, node2vec, and LINE, their results were obtained using combined patient-service and patient-specialty graphs. Additionally, we used both precision-recall AUC (PR-AUC) and AUC instead of micro- or macro-F1 to evaluate the performance for readmission prediction. For each method, the PR-AUC and AUC were averaged from the testing results of 10-fold cross-validation. Table 5 summarized the performance for readmission prediction, where it shows that ME2Vec outperformed all other methods in terms of both PR-AUC and AUC. Similar to the previous tasks, the graph embedding based methods showed consistently better performance than matrix factorization methods.

### Using ME2Vec as pretrained input embeddings for recurrent models

Recurrent neural networks (e.g., GRU and LSTM) have been widely adopted to model the long-range dependencies and nonlinear dynamics of sequential data. It has been the *de facto* approach to embed the individual tokens in a sequence into low-dimensional dense vectors before feeding them into recurrent models for enhanced performance, as embedding can better capture the relationship between input tokens than one-hot or multi-hot encoding. The weights of the input embedding layer can be randomly initialized and optimized together with the recurrent model in an end-to-end training, or initialized using pretrained embedding vectors and fine-tuned along with the recurrent model.

In this experiment, we evaluate the effectiveness of service embeddings from ME2Vec and several competitive baselines in two sequential learning tasks that (1) predicts the probabilities of patients diagnosed as CLL from their longitudinal EHR records, and (2) predicts ICU readmission from patients’ medical records. The design of the recurrent model used for this experiment was detailed in Supplementary Section B.2. For each patient in the IQVIA dataset, we prepared a sequence that tracks the patient’s received medical services in the temporal order to predict CLL diagnosis. Each sequence was either truncated or padded to be 400 in length. Specifically, we first randomly divided all the patients into three groups for training (80%), validating (10%), and testing (10%). The ratio of the positive (w/ CLL) versus negative (w/o CLL) was kept the same across the three groups through stratified split. Secondly, we run ME2Vec, metapath2vec, and word2vec on the training dataset and obtained the service embeddings. Thirdly, we trained four recurrent models using the training dataset, with one whose input embedding layer was randomly initialized and the others’ input embedding layers initialized using the pretrained ME2Vec, metapath2vec, and word2vec, respectively. Finally, we evaluated the models with the best validating performance on the testing datasets. We repeated the above procedures 10 times and reported the average prediction accuracy in PR-AUC. The testing procedures for the eICU dataset were similar, except that (1) the sequence for each admission comprises all the medical services administered during the admission; (2) in addition to the above three embedding algorithms, we also run node2vec on the eICU dataset; (3) both PR-AUC and AUC were reported.

**Table 6:** Averaged performance of the recurrent model predicting CLL diagnoses from patients’ service sequences.

	PR-AUC	Improvement
Input embedding initialized with pretrained ME2Vec	**0.823**	0%
Input embedding initialized with pretrained metapath2vec	0.766	+7.4%
Input embedding initialized with pretrained word2vec	0.759	+8.4%
Input embedding randomly initialized	0.753	+9.3%

Bolded numbers indicate best performance compared with the rest in the column.

**Table 7:** Averaged performance of the recurrent model predicting ICU readmission from patients’ service sequences.

	PR-AUC	AUC
Input embedding initialized with pretrained ME2Vec	0.204	**0.595**
Input embedding initialized with pretrained metapath2vec	**0.209**	0.586
Input embedding initialized with pretrained node2vec	0.194	0.569
Input embedding initialized with pretrained word2vec	0.181	0.563
Input embedding randomly initialized	0.173	0.562

Bolded numbers indicate best performance compared with the rest in the column.

As shown in Tables 6 and 7, on the IQVIA dataset, pretrained service embeddings using ME2Vec can substantially improve the prediction accuracy than random initialization (9.3%) and word2vec (8.4%); on the eICU dataset, ME2Vec and metapath2vec achieved comparable and consistently better performance on both PR-AUC and AUC than other methods. It is worth noting that in many NLP tasks, the performance improvement brought by using pretrained embeddings (or pretrained language models) is conditioned upon the access to large-scale, cheap, and unlabeled text corpora (e.g., Wikipedia or millions of web pages). However, such abundant data sources are usually not available in medical data analysis due to the legal and regulatory barriers to sharing patient-level data across different institutions. In this experiment, we show that the service embeddings given by ME2Vec can improve the performance of downstream tasks without requiring extra patient-level data. This advantage is primarily ascribed to that in the service embedding of ME2Vec, the original patient journeys are only used to construct the service graph and generate pseudo journeys via biased random walk instead of for the actual contextualized embedding learning process like in word2vec. Therefore, service embeddings from ME2Vec can generalize robustly to unseen patient journeys as long as the new patient journeys follow similar transition probabilities of medical services, which is a fundamental presumption in contextualized embedding and works well in practice.

## Discussion and conclusion

In this paper, we proposed ME2Vec, a graph-based, hierarchical medical entity embedding framework. ME2Vec offers a comprehensive set of functionalities for embedding medical services, doctors, and patients. We designed a time-aware service embedding that can leverage the temporal profiles of medical services to characterize their importance through random-walk based node embedding. We also adapted a recent state-of-the-art graph embedding algorithm, GAT, to learning doctor embeddings in an auxiliary task that can reflect their administered services and primary specialties. Moreover, we developed an effective and scalable approach of node embedding for attributed multigraph that uniquely addressed the difficulty of patient embedding learning from both doctors and services. We conducted a number of experiments on two real-world clinical datasets, including node classification, link prediction, readmission prediction, and pretraining input embeddings for sequential learning. The results showed consistent performance improvements of ME2Vec compared with strong baselines on different tasks, suggesting the potentials of ME2Vec as a comprehensive and general-purpose solution for representation learning of EHR data.

Concerns may exist regarding the order of the hierarchy in ME2Vec that embeds services first, then doctors, finally patients. In designing ME2Vec, the set of principles that we adhered to determined the order of embedding learning of medical entities and ruled out other possibilities. Specifically, the embedding of medical services is self-contained, and serves as the cornerstone of all other embeddings. It adheres to the principle that service embeddings should reflect the temporal distances between different medical services, such that a rare medical service can “stand out” in the embedding space. Doctor embeddings are calculated directly from service embeddings using the GAT model, adhering to the principle that the medical services performed by a doctor should reflect the doctor’s primary specialty. Finally, patient embeddings are calculated from both service and doctor embeddings, adhering to the principle that for any two patients, their embeddings should reflect their similarity in terms of shared doctors and medical services. Consequently, changing the order of embedding learning would imply a different set of principles, or more precisely, different understanding of the ways medical entities interact.

However, other options do not make sense as much as ME2Vec does. For example, if learning the patient and doctor embeddings from the patient-doctor bipartite graph as the first step, we can only focus on preserving the edges between patient and doctor nodes as there is no other information available at this point that can be leveraged or incorporated. In the next step of learning service embedding, we do not have much options but create a big graph that consists of all medical entities (for patient and doctor nodes their embeddings are known already) and derive service embedding, such that the relations of medical services with respect to patients and doctors can be preserved. As in both steps the learning criterion is to preserve graph structures, one would argue that a more reasonable and efficient solution is to simply create a big graph containing all medical entities from the very beginning and learn their embeddings simultaneously. In fact, this largely coincides with the option of using heterogeneous network embedding methods to learn all embeddings simultaneously that we have already discussed and compared to in the main body of the paper. Therefore, in the realm of hierarchical embeddings, the order of embedding learning adopted by ME2Vec represents the most or even the only reasonable option that can leverage all kinds of versatile information in EHR data via the set of principles proposed by us.

Although overall medical entities in EHR are heterogeneous, we make the embedding learning process homogeneous in each hierarchy by carefully designing entity-specific training paradigms tailored to the structural properties and statistical characteristics of entities. An alternative solution is to learn their embeddings altogether in one graph using heterogeneous network embedding techniques such as metapath2vec. One advantage of this approach is that the learned embeddings of heterogeneous nodes are in the same space, therefore their distances or similarities can be more easily evaluated. Additionally, it reduces the complexity of embedding algorithms by allowing to process various types of nodes/edges simultaneously. However, this is at the cost of restricted flexibility of designing entity-specific training paradigms where ME2Vec prevails. For example, for medical services we employ random walk based contextualized embedding to characterize their temporal profiles, whereas for doctor embeddings, we are not interested in the temporal information of administered services but their relations to primary specialties. These two types of learning are distinct in nature (unsupervised versus supervised) and data structures (one-dimensional context window versus non-Euclidean neighborhood over graphs), and thus difficult to be replaced by one unified paradigm without performance degradation. Another choice of learning heterogeneous nodes and edges together is knowledge graph embedding^43^, which allows customized processing of nodes/edge of unique types as well as joint and end-to-end learning. It is left as our future works to explore this direction.

## Supplementary information

Supplementary Information.

## Data Availability

The IQVIA dataset is proprietary and not publicly accessible, as the data sources are used for commercial purposes by IQVIA. The eICU dataset can be accessed at https://eicu-crd.mit.edu.
