# Supplementary material for: Leveraging graph-based hierarchical medical entity embedding for healthcare applications
Source: Sci Rep. 2021 Mar 12;11:5858. doi: 10.1038/s41598-021-85255-w (PMC7955058; doi:10.1038/s41598-021-85255-w)
Supplement: Supplementary file 1 — Supplementary Information. [file 41598_2021_85255_MOESM1_ESM.pdf]

Supplementary Online Information

Leveraging Graph-Based Hierarchical Medical Entity Embedding for Healthcare Applications

Tong Wu[1], Yunlong Wang[1], Yue Wang[1], Emily Zhao[1], and Yilian Yuan[1]

[1]Advanced Analytics, IQVIA Inc., Plymouth Meeting, Pennsylvania, USA, 19462

Corresponding Author:

Yunlong Wang
Advanced Analytics, IQVIA Inc.
1 IMS Dr., Plymouth Meeting, PA, USA, 19462
E-mail: Yunlong.Wang@iqvia.com
Tel: +1 (610) 729-3910

A Details of `ME2Vec`

A.1 Service Embedding

The details of learning medical service embedding vectors are given in Algorithm 1. The `combinations` function lists all the unique pairs of medical services within the segment $J_{seg}^{(i)}$. To embed medical services, we first obtain the adjacency matrix $\mathbf{A}_{svc}$ from patient journeys and use it to generate biased random walks, then optimize the embeddings of medical services by maximizing the probability of each service "seeing" its neighbors in the walks via stochastic gradient descent (SGD).

Algorithm 1 Medical service embedding

Input: Patient journeys $\{J^{(i)}\}_{i=1}^{P}$, context window length T, dimension p, walks per node r, walk length l, context size k

Output: Service embedding $\mathbf{S} \in \mathbb{R}^{|S| \times p}$

$\mathbf{A}_{svc} \leftarrow \mathbf{0} \in \mathbb{R}^{|S| \times |S|}$
for $i = 1$ **to** P **do**
  for $j = 1$ **to** $\lceil \frac{|J^{(i)}|}{T} \rceil$ **do**
    $J_{seg}^{(i)} \leftarrow J^{(i)}[(j-1)T : jT]$
    for s_x, s_y **in** `combinations`$(J_{seg}^{(i)}, 2)$ **do**
      $\mathbf{A}_{svc}[s_x, s_y]$ += 1
      $\mathbf{A}_{svc}[s_y, s_x]$ += 1
$E_{svc} \leftarrow \mathbf{A}_{svc}$, $G_{svc} \leftarrow \{S, E_{svc}\}$
$walks \leftarrow \{\}$
for $iter = 1$ **to** r **do**
  for all nodes $s \in S$ **do**
    $walk \leftarrow$ `BiasedRandomWalk`(G_{svc}, s, l)
    Append $walk$ to $walks$
$\mathbf{S} \leftarrow$ `SGD`$(k, p, walks)$
return $\mathbf{S}$

A.2 Doctor Embedding

In this work, we deployed two layers of graph attention networks to enhance the learning capability. The structure of the two-level doctor embedding model is shown in Figure 1.

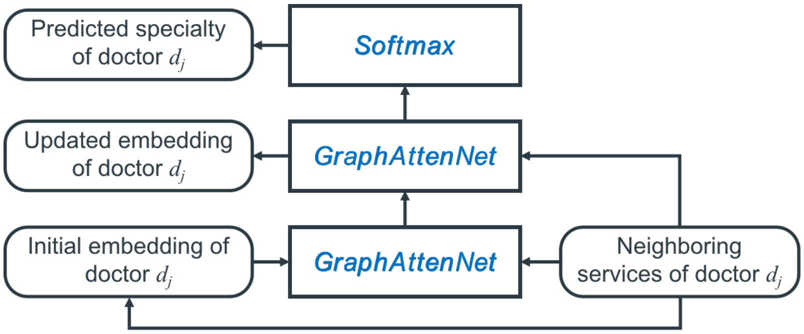

Figure 1. Structural diagram of a 2-level attention-based doctor embedding model. The embedding vectors of the medical services conducted by doctor d_j are pre-trained in the step of service embedding.

As the proposed auxiliary task is a supervised classification, we configure the final layer of the model as a `softmax`. In addition, the output embedding from each of the K attention heads of the second `GraphAttenNet` are averaged instead of concatenated, followed by the final `softmax`:

$$\mathbf{d}'_j = \sigma\left(\frac{1}{K}\sum_{k=1}^{K}\sum_{s_i \in \mathcal{N}_{d_j}} \alpha_{ij}^{k}\mathbf{W}^{k}\mathbf{s}_i\right). \tag{1}$$

We summarize the steps for doctor embedding in Algorithm 2, where `GraphAttenNet-2L` denotes the operations of two `GraphAttenNet` stacked together; `CrossEnt` denotes cross-entropy loss; L_{gt} and L_{pred} represent the ground-truth and the predicted doctor specialties, respectively.

Algorithm 2 Doctor embedding

Input: Patient journeys $\{J^{(i)}\}_{i=1}^{P}$, service embedding $\mathbf{S}$, number of attention heads K, learning rate η
Output: Doctor embedding $\mathbf{D} \in \mathbb{R}^{|D| \times p'}$, aggregation functions $\{\mathbf{W}^k, \mathbf{a}^k\}_{k=1}^{K}$

1: $L_{gt} \leftarrow D \leftarrow G_{doc} \leftarrow \{J^{(i)}\}_{i=1}^{P}$
2: **for all** node $d \in D$ **do**
3: $\quad \mathbf{d}_{init} \leftarrow \frac{1}{|N_d|}\sum_{i=1}^{|N_d|}\mathbf{s}_i \ (s_i \in N_d)$
4: $\mathbf{D}' \leftarrow$ `GraphAttenNet-2L`$(\mathbf{D}_{init}, \mathbf{S})$
5: $L_{pred} \leftarrow$ `softmax`$(\mathbf{D}')$
6: **while** `CrossEnt`(L_{pred}, L_{gt}) **is large do**
7: $\quad \mathbf{D}' \leftarrow$ `GraphAttenNet-2L`$(\mathbf{D}_{init}, \mathbf{S})$
8: $\quad L_{pred} \leftarrow$ `softmax`$(\mathbf{D}')$
9: $\quad \{\mathbf{W}^k, \mathbf{a}^k\} \leftarrow \{\mathbf{W}^k, \mathbf{a}^k\} - \eta\nabla($`CrossEnt`$(L_{pred}, L_{gt}))$
10: $\mathbf{D} \leftarrow$ `GraphAttenNet`$(\mathbf{D}_{init}, \mathbf{S})$
11: **return** $\mathbf{D}, \{\mathbf{W}^k, \mathbf{a}^k\}_{k=1}^{K}$

A.3 Patient Embedding

We summarize the steps for patient embedding in Algorithm 3.

Algorithm 3 Patient embedding

Input: Patient journeys $\{J^{(i)}\}_{i=1}^{P}$, service embedding $\mathbf{S}$, doctor embedding $\mathbf{D}$, learning rate η
Output: Patient embedding $\mathbf{P} \in \mathbb{R}^{P \times p''}$, linear transformation parameters $\{\mathbf{W}_a, \mathbf{b}_a\}$

1: $G_{pat} \leftarrow \{J^{(i)}\}_{i=1}^{P}$
2: $G_{pat} \leftarrow$ `Duplication_Annotation`$(G_{pat}, \mathbf{S}, \mathbf{D})$
3: **for all** node $p \in P$ **do**
4: $\quad p_2(h_{s_i,d_j}|p_k) = \frac{\exp(\mathbf{h}_{s_i,d_j} \cdot \mathbf{p}_k)}{\sum_{l \in \{h\}} \exp(\mathbf{h}_l \cdot \mathbf{p}_k)}$
5: $\quad \hat{p}_2(h_{s_i,d_j}|p_k) = \frac{w_{p_k \to h_{s_i,d_j}}}{\sum_{l \in N_{p_k}} w_{p_k \to h_l}}$
6: **while** $D_{KL}(\hat{p}_2 \parallel p_2)$ **is large do**
7: $\quad$ Algorithmic steps in lines 3–5
8: $\quad \{\mathbf{P}, \mathbf{W}_a, \mathbf{b}_a\} \leftarrow \{\mathbf{P}, \mathbf{W}_a, \mathbf{b}_a\} - \eta\nabla(D_{KL}(\hat{p}_2 \parallel p_2))$
9: **return** $\mathbf{P}, \{\mathbf{W}_a, \mathbf{b}_a\}$

Note that $D_{KL}(\hat{p}_2 \parallel p_2)$ denotes the *Kullback–Leibler* (*KL*) distance or divergence between $\hat{p}_2$ and p_2.

B Details of Experiments

B.1 Criteria of Choosing Patients into Negative Cohort

Following criteria are used for for generating the negative cohort: CLL risk factor, diagnosis, procedure, and prescription. A patient must meet at least three of the criteria before being included into the negative cohort. The risk factors include anemia, chills, fatigue, fever, night sweats and pain, Sjögren's syndrome, weakness, and weight loss. The related diagnoses include recurrent infection, Epstein-Barr infection, Helicobacter pylori infection, HIV/AIDS, Human T-lymphotrophic virus Type-I, rheumatoid arthritis, hypogammaglobulinemia, psoriasis, Wiskott-Aldrich syndrome, and pneumonia. The related procedures include tissue culture and chromosome analysis, increased frequency of CBC/ or blood test, and flow cytometry. The related prescriptions include Dexamethasone, Neupogen, and Prednisolone.

B.2 Using `ME2Vec` as Pretrained Input Embeddings for Recurrent Models

As shown in Figure 2, the recurrent model we used for this experiment is a two-layer LSTM with 256-dimensional hidden units and a 128-dimensional input embedding layer. The hidden outputs of the LSTM enter a global max-pooling layer that for each of the 256 dimensions, keeps the maximum value from all the time steps. The outputs of the global max-pooling layer are further processed by an multilayer perceptron (MLP) ended with a `sigmoid` function to make the final prediction. The recurrent model is trained for 30 epochs using an Adam optimizer with a batch size of 64 and learning rate of 1e–4.

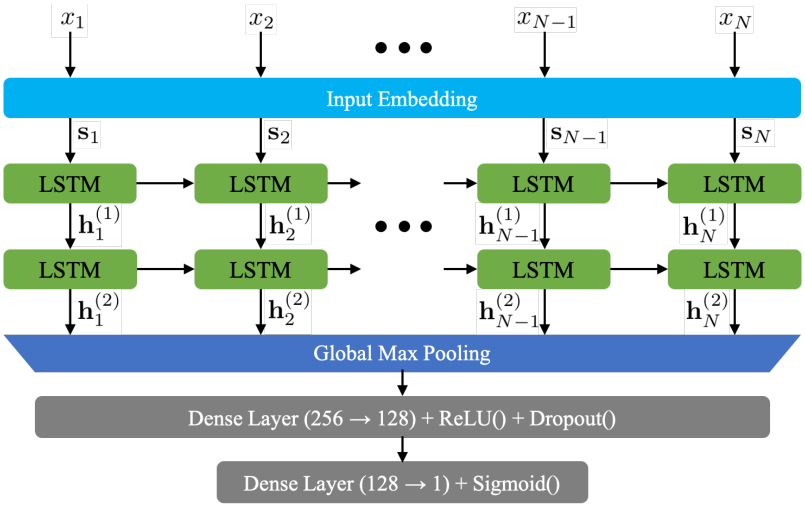

Figure 2. Structure of the recurrent model for predicting diagnosis results from patients' service sequences.
